# Dichlorido-2κ^2^ *Cl*-{μ-6,6′-dimeth­oxy-2,2′-[propane-1,3-diylbis(nitrilo­methyl­idyne)]diphenolato-1κ^4^ *O* ^1^,*N*,*N*′,*O* ^1′^:2κ^2^ *O* ^1^,*O* ^1′^}copper(II)zinc(II)

**DOI:** 10.1107/S1600536809006928

**Published:** 2009-03-06

**Authors:** Qingyun Liu, Shengsong Ge, Guangwen Cui

**Affiliations:** a School of Chemical & Environmental Engineering, Shandong University of Science and Technology, Qingdao 266510, People’s Republic of China

## Abstract

In the title compound, [CuZnCl_2_(C_19_H_20_N_2_O_4_)], the Cu^II^ ion exhibits a slightly distorted square-planar coordination geometry defined by two N atoms and two O atoms of the 6,6′-dimeth­oxy-2,2′-[propane-1,3-diylbis(nitrilo­methyl­idyne)]diphenolate Schiff base ligand. The Zn^II^ ion is also four-coordinated by the two phenolate O atoms of the Schiff base ligand and by two *cis*-coordinated chloride anions.

## Related literature

For the physical and chemical properties of heterometallic complexes, see: Ni *et al.* (2005 ▶, 2007 ▶); Ward (2007 ▶) and for their roles in biological systems, see: Karlin (1993 ▶). For bond-length data, see: Korupoju *et al.* (2000 ▶); Gheorghe *et al.* (2006 ▶). For the restraints used in the refinement, see: Ng (2005 ▶).
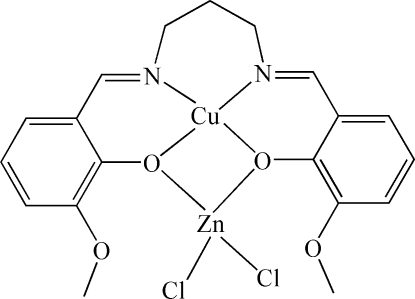


## Experimental

### 

#### Crystal data

[CuZnCl_2_(C_19_H_20_N_2_O_4_)]
*M* *_r_* = 540.18
Orthorhombic, 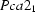

*a* = 13.0181 (9) Å
*b* = 10.8503 (8) Å
*c* = 14.7758 (11) Å
*V* = 2087.1 (3) Å^3^
*Z* = 4
Mo *K*α radiation
μ = 2.45 mm^−1^
*T* = 298 K
0.20 × 0.10 × 0.08 mm

#### Data collection

Bruker APEXII CCD area-detector diffractometer
Absorption correction: multi-scan (*SADABS*; Sheldrick, 2003 ▶) *T*_min_ = 0.744, *T*_max_ = 0.828
9818 measured reflections
3486 independent reflections
3084 reflections with *I* > 2σ(*I*)
*R*_int_ = 0.025

#### Refinement

*R*[*F* ^2^ > 2σ(*F* ^2^)] = 0.029
*wR*(*F* ^2^) = 0.074
*S* = 1.07
3486 reflections
262 parameters
13 restraints
H-atom parameters constrained
Δρ_max_ = 0.63 e Å^−3^
Δρ_min_ = −0.58 e Å^−3^
Absolute structure: Flack (1983 ▶), 1564 Friedel pairs
Flack parameter: 0.006 (15)

### 

Data collection: *APEX2* (Bruker, 2004 ▶); cell refinement: *SAINT-Plus* (Bruker, 2001 ▶); data reduction: *SAINT-Plus*; program(s) used to solve structure: *SHELXL97* (Sheldrick, 2008 ▶); program(s) used to refine structure: *SHELXL97* (Sheldrick, 2008 ▶); molecular graphics: *XP* in *SHELXTL* (Sheldrick, 2008 ▶); software used to prepare material for publication: *XP* in *SHELXTL*.

## Supplementary Material

Crystal structure: contains datablocks global, I. DOI: 10.1107/S1600536809006928/hg2482sup1.cif

Structure factors: contains datablocks I. DOI: 10.1107/S1600536809006928/hg2482Isup2.hkl

Additional supplementary materials:  crystallographic information; 3D view; checkCIF report

## Figures and Tables

**Fig. 1.**
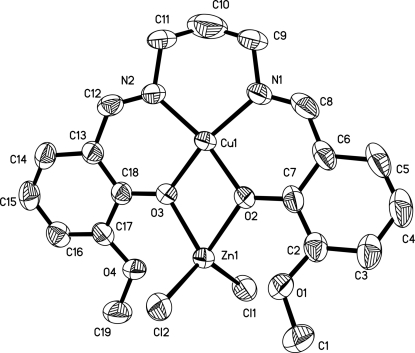
A view of (I) with the unique atom-labelling scheme. Displacement ellipsoids are drawn at the 30% probability level, all hydrogen atoms are ommited for clarity.

## supplementary crystallographic information

### Comment

Heterometallic complexes have been intensively focused on owing to their unique physical and chemical properties (Ward *et al.*, 2007; Ni *et al.*, 2005 and Ni *et al.* 2007). In addition, these compounds exist at the active sites of many metalloenzymes and play important roles in biological systems (Karlin, 1993). Whereas, it is necessary to further widen the system of heterometallic compounds. Herein, a new heterometallic dinuclear (Cu^II^Zn^II^) compound has been obtained. Its structure is depicted in the Figure 1.

Compound I is a dinuclear neutral complex with a slightly distorted planar configuration. The Cu^II^ atom is coordinated by two nitrogen atoms and two oxygen atoms from *L*^2-^ ligand forming a square-planar geometry. The coordination environment of each Zn^II^ atom is in a distorted tetrahedral geometry composed of two oxygen atoms from *L*^2-^ ligand and two chlorine atoms occupying the the other two positions. The dihedral angle of two aromatic rings is 26.90 (4)°. The Cu^II^ atom and Zn^II^ atom are connected *via* two bridging phenoxo oxygen atoms of *L*^2-^ ligand, The bond lengths of Cu—O, Cu—N, Zn—O and Zn—Cl are normal (Gheorghe *et al.* 2006 and Korupoju *et al.*, 2000).

### Experimental

The H_2_L ligand and complex CuL was synthesized according to the previous literature (Gheorghe *et al.* 2006). the synthesis method of the compound I was obtained by allowing a mixure of CuL (0.088 g, 0.2 mmol) and ZnCl_2_.2H_2_O(0.044 g, 0.2 mmol) to be stirred in the methanol solution at room temperature, cooled down to room temperature and then filtered. Suitable yellow needle-shaped crystals were obtained *via* slow evaporation of the filtrate at room temperature.

### Refinement

All H-atoms bound to carbon were refined using a riding model with distance C—H = 0.93 Å, *U*_iso_ = 1.2*U*_eq_ (C) for aromatic atoms and C—H = 0.96 Å, *U*_iso_ = 1.5*U*_eq_ (C) for methyl atoms. and the 'isor' order is used to restrain the C10 atom with *wARP* as 0.005 (Ng, 2005).

### Crystal data

**Table:**

[CuZnCl_2_(C_19_H_20_N_2_O_4_)]	*F*(000) = 1092
*M**_r_* = 540.18	*D*_x_ = 1.719 Mg m^−^^3^
Orthorhombic, *P**c**a*2_1_	Mo *K*α radiation, λ = 0.71073 Å
Hall symbol: P 2c -2ac	Cell parameters from 4074 reflections
*a* = 13.0181 (9) Å	θ = 2.5–26.1°
*b* = 10.8503 (8) Å	µ = 2.45 mm^−^^1^
*c* = 14.7758 (11) Å	*T* = 298 K
*V* = 2087.1 (3) Å^3^	Needle, green
*Z* = 4	0.20 × 0.10 × 0.08 mm

### Data collection

**Table:**

Bruker APEXII CCD area-detector diffractometer	3486 independent reflections
Radiation source: fine-focus sealed tube	3084 reflections with *I* > 2σ(*I*)
graphite	*R*_int_ = 0.025
Detector resolution: 0 pixels mm^-1^	θ_max_ = 25.0°, θ_min_ = 1.9°
φ and ω scans	*h* = −15→15
Absorption correction: multi-scan (*SADABS*; Sheldrick, 2003)	*k* = −11→12
*T*_min_ = 0.744, *T*_max_ = 0.828	*l* = −17→15
9818 measured reflections	

### Refinement

**Table:**

Refinement on *F*^2^	Secondary atom site location: difference Fourier map
Least-squares matrix: full	Hydrogen site location: inferred from neighbouring sites
*R*[*F*^2^ > 2σ(*F*^2^)] = 0.029	H-atom parameters constrained
*wR*(*F*^2^) = 0.074	*w* = 1/[σ^2^(*F*_o_^2^) + (0.0389*P*)^2^ + 0.0971*P*] where *P* = (*F*_o_^2^ + 2*F*_c_^2^)/3
*S* = 1.07	(Δ/σ)_max_ = 0.001
3486 reflections	Δρ_max_ = 0.63 e Å^−^^3^
262 parameters	Δρ_min_ = −0.58 e Å^−^^3^
13 restraints	Absolute structure: Flack (1983), 1564 Friedel pairs
Primary atom site location: structure-invariant direct methods	Flack parameter: 0.006 (15)

### Special details

**Table:**

Geometry. All e.s.d.'s (except the e.s.d. in the dihedral angle between two l.s. planes) are estimated using the full covariance matrix. The cell e.s.d.'s are taken into account individually in the estimation of e.s.d.'s in distances, angles and torsion angles; correlations between e.s.d.'s in cell parameters are only used when they are defined by crystal symmetry. An approximate (isotropic) treatment of cell e.s.d.'s is used for estimating e.s.d.'s involving l.s. planes.
Refinement. Refinement of *F*^2^ against ALL reflections. The weighted *R*-factor *wR* and goodness of fit *S* are based on *F*^2^, conventional *R*-factors *R* are based on *F*, with *F* set to zero for negative *F*^2^. The threshold expression of *F*^2^ > σ(*F*^2^) is used only for calculating *R*-factors(gt) *etc*. and is not relevant to the choice of reflections for refinement. *R*-factors based on *F*^2^ are statistically about twice as large as those based on *F*, and *R*- factors based on ALL data will be even larger.

### Fractional atomic coordinates and isotropic or equivalent isotropic displacement parameters (Å^2^)

**Table:**

	*x*	*y*	*z*	*U*_iso_*/*U*_eq_	
Zn1	0.59914 (3)	0.74035 (3)	0.69902 (3)	0.04337 (13)	
Cu1	0.59183 (3)	1.03456 (3)	0.71028 (4)	0.04411 (14)	
Cl2	0.71295 (8)	0.63787 (10)	0.78281 (9)	0.0590 (3)	
Cl1	0.48653 (8)	0.64556 (11)	0.60882 (10)	0.0653 (4)	
O1	0.4768 (2)	0.6796 (3)	0.8322 (2)	0.0607 (8)	
O4	0.7243 (2)	0.7042 (2)	0.5679 (2)	0.0531 (7)	
O3	0.6649 (2)	0.9031 (2)	0.6508 (2)	0.0468 (7)	
C6	0.3763 (3)	0.9888 (5)	0.8239 (3)	0.0537 (12)	
N1	0.4874 (3)	1.1465 (3)	0.7582 (3)	0.0596 (10)	
O2	0.5262 (2)	0.8915 (2)	0.76318 (19)	0.0442 (7)	
C12	0.7778 (4)	1.1331 (3)	0.6360 (3)	0.0486 (10)	
H12	0.8230	1.1989	0.6294	0.058*	
C15	0.9470 (4)	0.9069 (4)	0.5223 (3)	0.0557 (11)	
H15	1.0118	0.9054	0.4958	0.067*	
C13	0.8147 (3)	1.0147 (3)	0.6042 (3)	0.0421 (9)	
C7	0.4389 (3)	0.8850 (4)	0.8080 (3)	0.0445 (10)	
C17	0.7914 (3)	0.8007 (4)	0.5642 (3)	0.0439 (9)	
C5	0.2819 (3)	0.9734 (5)	0.8696 (3)	0.0650 (13)	
H5	0.2399	1.0415	0.8793	0.078*	
C18	0.7549 (3)	0.9076 (3)	0.6083 (3)	0.0388 (8)	
C1	0.4576 (4)	0.5620 (4)	0.8709 (5)	0.0845 (18)	
H1A	0.5132	0.5074	0.8566	0.127*	
H1B	0.3947	0.5293	0.8469	0.127*	
H1C	0.4518	0.5700	0.9354	0.127*	
C3	0.3144 (3)	0.7587 (5)	0.8888 (3)	0.0633 (13)	
H3	0.2943	0.6824	0.9113	0.076*	
C16	0.8847 (3)	0.8003 (4)	0.5211 (3)	0.0534 (11)	
H16	0.9069	0.7298	0.4910	0.064*	
C11	0.6734 (4)	1.2928 (3)	0.6876 (5)	0.0794 (15)	
H11A	0.7012	1.3144	0.7465	0.095*	
H11B	0.7113	1.3392	0.6425	0.095*	
C8	0.4053 (4)	1.1105 (4)	0.7987 (4)	0.0592 (13)	
H8	0.3586	1.1722	0.8135	0.071*	
C19	0.7605 (4)	0.5875 (4)	0.5382 (4)	0.0653 (13)	
H19A	0.7066	0.5276	0.5440	0.098*	
H19B	0.8181	0.5630	0.5747	0.098*	
H19C	0.7814	0.5930	0.4761	0.098*	
C2	0.4077 (3)	0.7705 (4)	0.8442 (3)	0.0506 (11)	
N2	0.6914 (3)	1.1585 (3)	0.6717 (3)	0.0499 (9)	
C10	0.5740 (5)	1.3279 (5)	0.6847 (5)	0.0981 (17)	
H10A	0.5504	1.3104	0.6237	0.118*	
H10B	0.5739	1.4168	0.6909	0.118*	
C9	0.4950 (4)	1.2824 (4)	0.7450 (6)	0.098 (3)	
H9A	0.4292	1.3114	0.7226	0.118*	
H9B	0.5056	1.3199	0.8038	0.118*	
C4	0.2511 (4)	0.8598 (5)	0.8999 (3)	0.0700 (14)	
H4	0.1877	0.8508	0.9280	0.084*	
C14	0.9123 (3)	1.0109 (5)	0.5619 (3)	0.0509 (11)	
H14	0.9530	1.0813	0.5615	0.061*	

### Atomic displacement parameters (Å^2^)

**Table:**

	*U*^11^	*U*^22^	*U*^33^	*U*^12^	*U*^13^	*U*^23^
Zn1	0.0359 (2)	0.0315 (2)	0.0628 (3)	0.00014 (17)	0.0017 (2)	−0.0053 (2)
Cu1	0.0362 (2)	0.0294 (2)	0.0667 (3)	0.00258 (17)	−0.0002 (3)	−0.0082 (3)
Cl2	0.0442 (6)	0.0572 (6)	0.0758 (8)	0.0039 (5)	−0.0013 (5)	0.0129 (6)
Cl1	0.0448 (6)	0.0532 (7)	0.0980 (9)	0.0004 (5)	−0.0096 (6)	−0.0273 (6)
O1	0.0527 (18)	0.0442 (17)	0.085 (2)	−0.0085 (14)	0.0185 (16)	−0.0066 (16)
O4	0.0498 (17)	0.0346 (14)	0.075 (2)	0.0015 (13)	0.0136 (15)	−0.0052 (14)
O3	0.0392 (16)	0.0337 (13)	0.0674 (19)	−0.0005 (11)	0.0092 (14)	−0.0024 (12)
C6	0.039 (2)	0.065 (3)	0.057 (3)	0.009 (2)	0.001 (2)	−0.026 (2)
N1	0.042 (2)	0.0386 (19)	0.099 (3)	0.0044 (15)	−0.0035 (19)	−0.0215 (18)
O2	0.0354 (15)	0.0364 (15)	0.0608 (18)	0.0018 (11)	0.0114 (13)	−0.0094 (12)
C12	0.055 (3)	0.035 (2)	0.055 (2)	−0.0055 (18)	−0.009 (2)	0.0120 (18)
C15	0.040 (2)	0.073 (3)	0.054 (3)	0.002 (2)	0.011 (2)	0.007 (2)
C13	0.037 (2)	0.043 (2)	0.046 (2)	0.0038 (17)	−0.0034 (17)	0.0089 (18)
C7	0.032 (2)	0.052 (3)	0.049 (2)	0.0009 (17)	0.0005 (18)	−0.0173 (18)
C17	0.041 (2)	0.045 (2)	0.046 (2)	0.0089 (18)	0.0035 (18)	0.0039 (18)
C5	0.044 (3)	0.086 (4)	0.064 (3)	0.016 (3)	0.008 (2)	−0.031 (3)
C18	0.034 (2)	0.042 (2)	0.040 (2)	0.0014 (17)	−0.0036 (17)	0.0060 (17)
C1	0.080 (4)	0.046 (3)	0.128 (5)	−0.015 (3)	0.030 (4)	−0.007 (3)
C3	0.050 (3)	0.084 (3)	0.056 (3)	−0.014 (2)	0.016 (2)	−0.019 (3)
C16	0.054 (3)	0.058 (3)	0.048 (3)	0.006 (2)	0.010 (2)	0.000 (2)
C11	0.071 (3)	0.0296 (19)	0.138 (4)	0.0002 (19)	−0.006 (3)	−0.009 (3)
C8	0.049 (3)	0.053 (3)	0.075 (3)	0.017 (2)	−0.009 (2)	−0.031 (2)
C19	0.077 (3)	0.044 (2)	0.075 (3)	0.002 (2)	0.015 (3)	−0.004 (2)
C2	0.040 (2)	0.059 (3)	0.052 (3)	−0.0037 (19)	0.0075 (19)	−0.020 (2)
N2	0.0406 (18)	0.0318 (15)	0.077 (2)	−0.0013 (14)	−0.0081 (17)	−0.0010 (16)
C10	0.102 (3)	0.050 (2)	0.142 (4)	0.014 (2)	−0.011 (3)	−0.001 (3)
C9	0.058 (3)	0.035 (2)	0.201 (8)	0.011 (2)	0.000 (4)	−0.031 (3)
C4	0.051 (3)	0.097 (4)	0.062 (3)	−0.005 (3)	0.021 (2)	−0.022 (3)
C14	0.039 (2)	0.060 (3)	0.054 (3)	−0.006 (2)	0.0008 (19)	0.018 (2)

### Geometric parameters (Å, °)

**Table:**

Zn1—O3	2.088 (3)	C7—C2	1.412 (6)
Zn1—O2	2.119 (2)	C17—C16	1.371 (5)
Zn1—Cl2	2.2280 (12)	C17—C18	1.413 (5)
Zn1—Cl1	2.2324 (12)	C5—C4	1.371 (6)
Cu1—O3	1.926 (3)	C5—H5	0.9300
Cu1—O2	1.936 (3)	C1—H1A	0.9600
Cu1—N2	1.953 (3)	C1—H1B	0.9600
Cu1—N1	1.956 (4)	C1—H1C	0.9600
O1—C2	1.347 (5)	C3—C2	1.388 (6)
O1—C1	1.421 (6)	C3—C4	1.381 (6)
O4—C17	1.364 (5)	C3—H3	0.9300
O4—C19	1.421 (5)	C16—H16	0.9300
O3—C18	1.330 (5)	C11—C10	1.349 (7)
C6—C7	1.409 (6)	C11—N2	1.495 (5)
C6—C5	1.412 (7)	C11—H11A	0.9700
C6—C8	1.423 (7)	C11—H11B	0.9700
N1—C8	1.286 (6)	C8—H8	0.9300
N1—C9	1.490 (6)	C19—H19A	0.9600
O2—C7	1.318 (5)	C19—H19B	0.9600
C12—N2	1.272 (5)	C19—H19C	0.9600
C12—C13	1.450 (5)	C10—C9	1.448 (9)
C12—H12	0.9300	C10—H10A	0.9700
C15—C14	1.350 (6)	C10—H10B	0.9700
C15—C16	1.413 (6)	C9—H9A	0.9700
C15—H15	0.9300	C9—H9B	0.9700
C13—C18	1.400 (5)	C4—H4	0.9300
C13—C14	1.417 (6)	C14—H14	0.9300
			
O3—Zn1—O2	71.43 (10)	H1A—C1—H1B	109.5
O3—Zn1—Cl2	109.81 (8)	O1—C1—H1C	109.5
O2—Zn1—Cl2	115.82 (9)	H1A—C1—H1C	109.5
O3—Zn1—Cl1	117.09 (9)	H1B—C1—H1C	109.5
O2—Zn1—Cl1	109.24 (8)	C2—C3—C4	120.3 (5)
Cl2—Zn1—Cl1	122.58 (4)	C2—C3—H3	119.8
O3—Cu1—O2	78.97 (10)	C4—C3—H3	119.8
O3—Cu1—N2	92.81 (13)	C17—C16—C15	120.0 (4)
O2—Cu1—N2	164.28 (13)	C17—C16—H16	120.0
O3—Cu1—N1	165.52 (14)	C15—C16—H16	120.0
O2—Cu1—N1	92.58 (14)	C10—C11—N2	114.8 (4)
N2—Cu1—N1	98.00 (15)	C10—C11—H11A	108.6
C2—O1—C1	119.1 (4)	N2—C11—H11A	108.6
C17—O4—C19	117.3 (3)	C10—C11—H11B	108.6
C18—O3—Cu1	128.6 (2)	N2—C11—H11B	108.6
C18—O3—Zn1	123.6 (2)	H11A—C11—H11B	107.5
Cu1—O3—Zn1	105.55 (12)	N1—C8—C6	128.6 (4)
C7—C6—C5	119.2 (5)	N1—C8—H8	115.7
C7—C6—C8	123.0 (4)	C6—C8—H8	115.7
C5—C6—C8	117.7 (4)	O4—C19—H19A	109.5
C8—N1—C9	114.7 (4)	O4—C19—H19B	109.5
C8—N1—Cu1	123.9 (3)	H19A—C19—H19B	109.5
C9—N1—Cu1	121.4 (3)	O4—C19—H19C	109.5
C7—O2—Cu1	128.8 (2)	H19A—C19—H19C	109.5
C7—O2—Zn1	124.7 (2)	H19B—C19—H19C	109.5
Cu1—O2—Zn1	104.01 (11)	O1—C2—C3	125.5 (4)
N2—C12—C13	128.2 (4)	O1—C2—C7	113.7 (3)
N2—C12—H12	115.9	C3—C2—C7	120.8 (4)
C13—C12—H12	115.9	C12—N2—C11	114.5 (4)
C14—C15—C16	119.8 (4)	C12—N2—Cu1	123.9 (3)
C14—C15—H15	120.1	C11—N2—Cu1	121.4 (3)
C16—C15—H15	120.1	C11—C10—C9	124.4 (6)
C18—C13—C14	119.6 (4)	C11—C10—H10A	106.2
C18—C13—C12	122.5 (4)	C9—C10—H10A	106.2
C14—C13—C12	117.7 (4)	C11—C10—H10B	106.2
O2—C7—C6	122.6 (4)	C9—C10—H10B	106.2
O2—C7—C2	119.1 (3)	H10A—C10—H10B	106.4
C6—C7—C2	118.3 (4)	C10—C9—N1	117.7 (5)
O4—C17—C16	125.7 (4)	C10—C9—H9A	107.9
O4—C17—C18	113.3 (3)	N1—C9—H9A	107.9
C16—C17—C18	121.0 (4)	C10—C9—H9B	107.9
C4—C5—C6	121.1 (4)	N1—C9—H9B	107.9
C4—C5—H5	119.4	H9A—C9—H9B	107.2
C6—C5—H5	119.4	C5—C4—C3	120.0 (4)
O3—C18—C13	122.7 (3)	C5—C4—H4	120.0
O3—C18—C17	119.0 (3)	C3—C4—H4	120.0
C13—C18—C17	118.3 (4)	C15—C14—C13	121.1 (4)
O1—C1—H1A	109.5	C15—C14—H14	119.5
O1—C1—H1B	109.5	C13—C14—H14	119.5
